# The Origin of Anomalous Density Behavior of Silica Glass

**DOI:** 10.3390/ma16186218

**Published:** 2023-09-15

**Authors:** Shangcong Cheng

**Affiliations:** Molecular Foundry of Lawrence Berkeley National Laboratory, Berkeley, CA 94720, USA; shangcongcheng@lbl.gov

**Keywords:** silica glass, anomalous density, medium-range ordering, nanoflake model, covalent bond, van der Waals bond

## Abstract

The anomalous density–temperature relationship of vitreous silica with low hydroxyl content is explained by the formation of medium-range ordering structure in the glass transition process. The ordered medium-range structure has the shape of a “nanoflake” and consists of two layers of SiO_4_ tetrahedra, bonded by O atoms located in the middle of the structure. The nanoflakes interact with their surrounding structures through both covalent chemical bonds and van der Waals bonds. In the formation of the van der Waals bonds, the orientation of SiO_4_ tetrahedra can change, which results in an increase in distance between the nanoflakes and their surrounding structures. Thus, there is a slight volume enlargement associated with the formation of nanoflakes. Since the nanoflakes’ formation starts at a temperature near 1480 °C, and the population of the nanoflakes grows continuously as temperature decreases until about 950 °C, the bulk volume of silica glass increases in the temperature range from about 1480 °C to 950 °C. Therefore, the density anomaly of silica glass can be explained as a byproduct of forming of medium-range ordering structure in the silica glass transition.

## 1. Introduction

Silica glass is the most essential glass-forming material with several technologically important properties. The structure, formation and properties of silica glass have been studied for many decades. One particular area of focus is its anomalous density–temperature relation. For most materials, the density increases as the material is cooled. In contrast, the density of silica glass decreases in a wide temperature range as it cools down from the high-temperature liquid state to the solid state [1,2,3,4,5,6]. Water also has a similar well-known anomalous density behavior: at a pressure of 1 atm, water has its density maximum at 4 °C. It has been believed that the density anomalies of both SiO_2_ and H_2_O are related to an expanded tetrahedral network structure as the materials cool down. The details of the structural variation are clearly critical to the understanding of the materials properties. In the past few decades, the structural origins of such anomalies have been intensively investigated through experiments and simulations [7,8,9,10,11,12,13]. Despite tremendous efforts, the structural origins of these density anomalies remain elusive.

This work aims to use the recently proposed nanoflake model to explain the anomalies found in the density–temperature relation of silica glass. The nanoflake model differs from the popular continuous random network (CRN) theory in that it emphasizes the medium-range ordering structure formed in the glass cooling process. Since the model was proposed a few years ago [14,15]. it has successfully explained various glass properties, including the viscosity behaviors in a wide temperature range, the step change in heat capacity, and the mechanical strength and brittleness of silica glass [16]. In this work the nanoflake model is utilized here to provide a structural explanation for the anomalies of the density–temperature relation of silica glass.

## 2. Anomalies of the Density–Temperature Relation of Silica Glass

**Figure 1:**
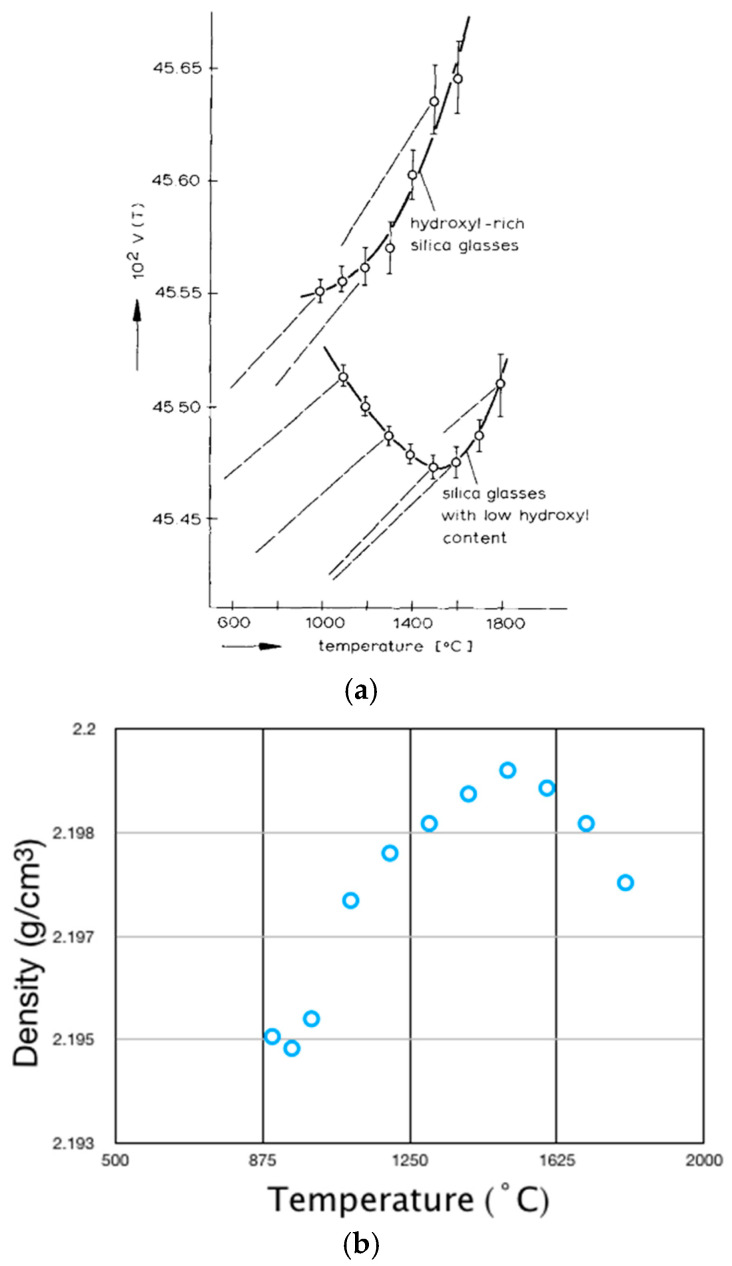
(**a**) Volume–temperature relation of silica glass. The vertical axis V can be converted as the reciprocal function of density. The top curve shows the normal density–temperature relation of glasses with high hydroxyl content. The bottom curve shows the abnormal density–temperature relation of glasses with low hydroxyl content in the temperature range from 1500 down to 1000 °C. Reprinted from reference [2]. (**b**) Density of silica glass as a function of temperature. The density maximum and minimum are located at around 1500 °C and 950 °C.

The classical experimental data on the density anomalies of silica glass were published about half a century ago by R. Bruckner, which is shown in Figure 1a [1,2]. Converting the volume axis V in Figure 1a to the reciprocal function of density, the figure shows the normal and abnormal characteristic density–temperature relations in the range of 1000–1800 °C for two kinds of vitreous silica glass. For glasses rich in hydroxyl content (higher than 1200 ppm), the density–temperature relation is normal (the top curve): the density increases as temperature decreases. For glasses with low hydroxyl content (less than 300 ppm), the density–temperature relation is abnormal (the bottom curve): there is a maximum density near 1500 °C, and from 1500 °C down to about 1000 °C the density does not increase but anomalously decreases. The anomalous density–temperature relationship of vitreous silica has been confirmed by several other experimental studies. The high-resolution density relaxation experiments by S. Sen et al. show that the volume vs. temperature behavior of silica glass changes from abnormal to normal at 950 °C, the temperature of the density minimum [3,4,5,6]. Although there were debates about the existence of the density maximum and the exact temperatures of the density maximum and minimum, the experimental results obtained from various techniques basically agree that the density maximum and minimum are located at around 1500 °C and 950 °C, as shown as Figure 1b [5].

## 3. Nanoflake Model for the Silica Glass’s Structure in the Medium Range

The nanoflake model for the medium-range ordering structure in silica glass has recently been proposed in the studies of the formation process and properties of silica glass [14,15]. The identification of two different temperature regions in the glass transition process is one of the key pieces of knowledge obtained from these studies. These two temperature regions are connected by the critical temperature Tc. For pure silica glass, this is the polymorphic inversion temperature of 1470 °C between crystal β-tridymite and β-cristobalite [17].

**Figure 2:**
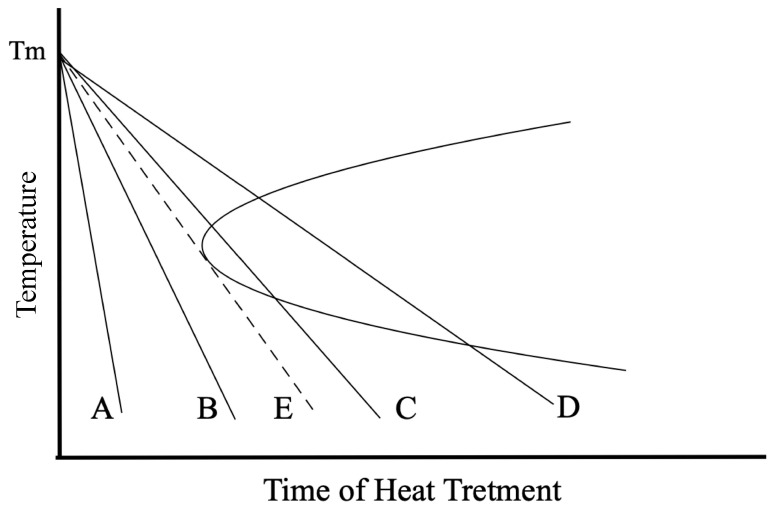
The time–temperature–transformation (T-T-T) curve and lines representing silica-forming processes with various cooling rates. The critical process E has the minimum cooling rate to avert crystallization. A and B processes with cooling rates above the critical one yield silica glass while C and D with cooling rates below the critical one yield polycrystalline silica.

It is known that the cooling rate is decisive for forming silica glass or cristobalite crystal. The cooling process for silica glass can be explained using the time–temperature–transformation (T-T-T) curve and lines representing different cooling processes, as shown in Figure 2. Since cooling processes crossing the solid T-T-T curve generate crystallization, a tangent line to the “nose” of the T-T-T curve is defined as the critical process (the dashed line E), which has the minimum required cooling rate to avert crystallization. The A and B processes have their cooling rates above the critical one and yield silica glass, whereas the C and D processes have cooling rates below the critical one and yield polycrystalline silica, whose particle sizes are also dependent on the cooling rate. Compared with the D process, the C process with a higher cooling rate is expected to generate crystal particles with smaller sizes than the D process. When a rising cooling rate approaches the critical one, the minimum value of the crystals’ average size will be gradually reached. 

**Figure 3:**
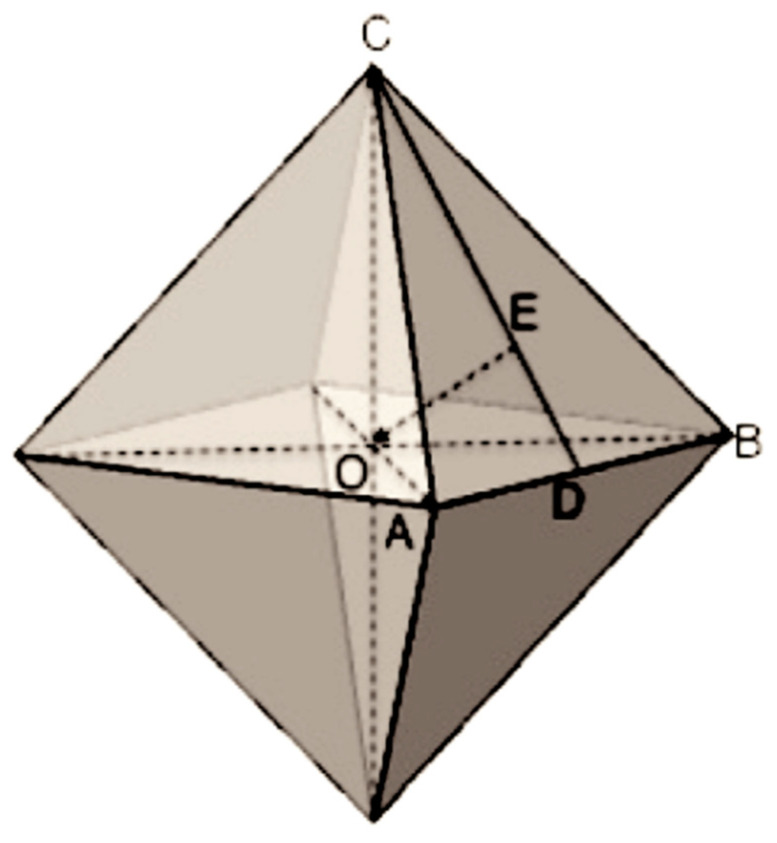
Schematic figure of octahedral cristobalite nucleus. Triangle ABC represents the facet of the crystal perpendicular to the [111] direction, the line OE.

**Figure 4:**
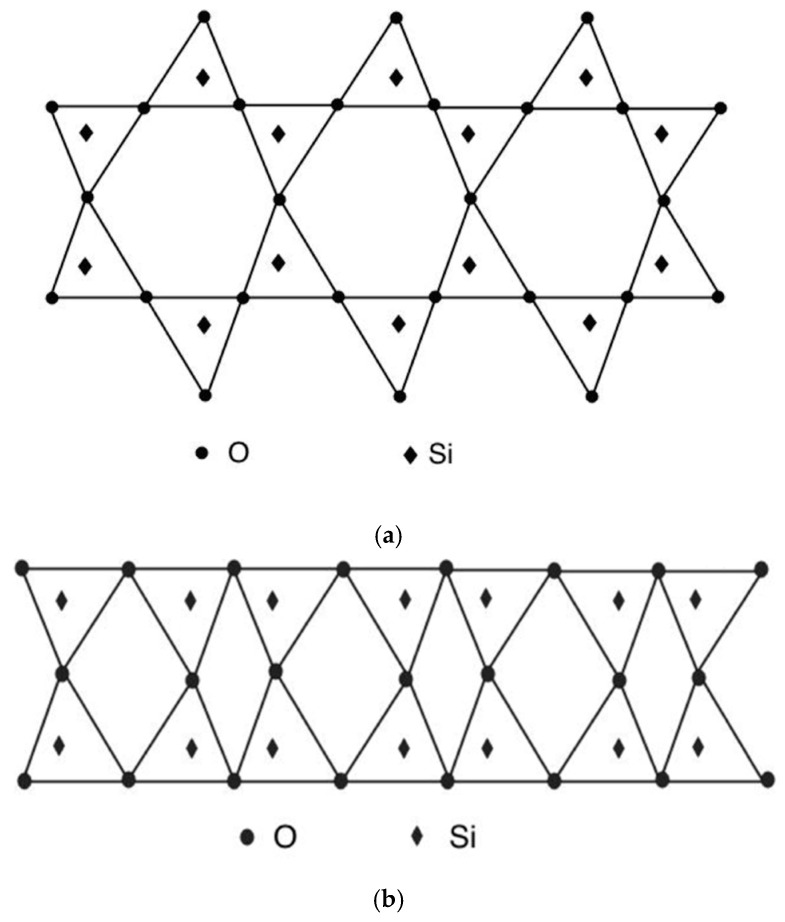
(**a**) Side view of facet structure of β-cristobalite nucleus. (**b**) Side view of the nanoflake.

β-cristobalite crystal at the early forming stage is octahedral in shape. Because the smallest crystal (crystalline nucleus) and a large crystal have the same three-dimensional structure, the smallest β-cristobalite crystal should have the structure shown in Figure 3 [14]. In the direction perpendicular to the facets of the nuclei particles, the structure is a two-dimensional crystal, where all Si-O bonds form six-membered rings. This is the same structure as the (111) plane of β-cristobalite. The side view of the facets is shown in Figure 4a. The two-layered structure in Figure 4a has a thickness of about 0.8 nm. The oxygen atom on the top layer in Figure 4a only bonds with one Si atom in the structure. As the crystal nucleus grows, it will bond with another Si atom in a new layer. However, the cooling rate of processes A and B is high, the embryonic clusters may enter the low-temperature zone before becoming crystal nuclei; the various membered rings on the facets of embryonic clusters have not yet organized into six-membered rings. Although in the lower temperature region, the embryonic clusters’ pathway to β-cristobalite nuclei is blocked, the structural evolution is still governed by thermal dynamics, and a one-dimensional ordering structure on the clusters’ facets called “nanoflake” is formed [14,18]. Looking along the direction perpendicular to the facets of the embryonic particle, the structure is not organized into regular six-membered rings. Instead, it consists of various membered rings. This is exactly the same as Zachariasen’s continuous random network theory depicts [19,20,21]. Figure 4b shows the side view of the nanoflakes. It can be seen that there is an oxygen atom layer in the middle of the structure, and the top and bottom layers of SiO_4_ tetrahedra are connected through this oxygen layer. The nanoflake is a one-dimensional ordering structure formed in the low-temperature region after adjusting for the orientation of the SiO_4_ tetrahedra on the top-most and bottom-most layers. The shape of clusters is approximately octahedral with edges about 1.9 nm in length. The clusters are randomly orientated and are also randomly spread in the structure. Thus, the volume isotropy of the system is maintained. Silicon–oxygen bonds and van der Waals bonds connect the clusters with outside structures. The silicon–oxygen bonds may be provided by oxygen atoms located on the edges and vertexes of the clusters. Oxygen atoms not situated on the borders may interact with outside structures by the van der Waals force, which is significantly weaker than chemical bonding [22]. Van der Waals bonds create spaces in the neighborhoods of nanoflakes where there are no silicon–oxygen bonds to connect the facets with the structures outside the clusters.

## 4. Explanation of Density Anomaly of Silica Glass

The structural model and its formation process described in the previous section demonstrate that the formation of nanoflakes not only brings a one-dimensional medium-range ordering structure into silica glass but also simultaneously creates regions where covalent oxygen silicon bonds in the neighborhood of nanoflakes are broken and replaced by van der Waals bonds. Since the interaction of van der Waals bond is much weaker and its interaction range larger than that of the covalent bond, the distances between nanoflakes and their surrounding structures must be increased where van der Waals bonds form [22]. Thus, as the population of the nanoflakes in the system increases, the volume of the vitreous silica glass also gradually increases. The slight volume enlargement in the formation of each nanoflake and the increase in nanoflake’s population as the temperature decreases from 1480 °C to 950 °C correlates exactly with the anomalous density behavior of silica glass. Hence, the density anomalies of silica glass can be explained by and support the theory of the medium-range ordering structure in the glass transition process.

One recognized feature of silica glass is the dependence of its properties on the cooling rate. Cooling rates in the high-temperature region from melting temperature Tm to the critical temperature Tc influence the total number of embryonic clusters. The number of clusters formed varies inversely with the cooling rate. Thus, the population of clusters in slow-cooled glass is more than that in fast-cooled glass, and the slow-cooled glass needs a wider temperature range to convert all clusters to more a stabilized structure. Accordingly, the temperature for the density minimum of silica glass with slow cooling is expected to be lower than that of fast-cooling glass. 

## 5. Discussion and Conclusions

Experimental data in Figure 1a indicate that normal or abnormal characteristic density–temperature relations of silica glasses are dependent on the hydroxyl content of glasses. A successful theory must explain this difference, i.e., why the density–temperature relation of silica glass with high hydroxyl content is normal whereas that of silica glass with low hydroxyl content is abnormal. 

As discussed in Section 3, embryonic clusters in silica with a cooling rate higher than the critical one may enter the low-temperature zone before becoming crystal nuclei. Although in the lower temperature region, the embryonic clusters’ pathway to β-cristobalite nuclei is blocked, its structural evolution is still governed by thermal dynamics. For silica with low hydroxyl, nanoflakes are formed in the cooling process after adjusting for the orientation of SiO_4_ tetrahedra on the top-most and bottom-most layers. The nanoflake formation creates spaces in the neighborhoods where physical silicon–oxygen bonds are replaced by van der Waals bonds.

The situation for silica glass with high hydroxyl is different. A hydrogen atom has one electron in its orbit and can either gain or lose an electron to form a stable outer electron shell, giving it a valency of +1 or −1. The additional hydrogen ions in the silica glass can bond with oxygen ions on the surface of the two-layer structures to form a stable structure. Thus, hydrogen ions can prevent the orientation change of SiO_4_ tetrahedra on the top surface of facets and deter the formation of the van der Waals bonds in the neighborhood of nanoflakes. Therefore, the volume of the silica glass with rich hydroxyl does not increase as temperature decreases, and the anomalous density–temperature relationship only applies to silica glasses with low hydroxyl content. In sodium silicate glass and other alkali silicate glasses, the alkali elements act in a similar way as hydrogen ions to prevent the formation of van der Waals bonds in the neighborhood of nanoflakes. Thus, the density–temperature relations of these silicate glasses are also normal.

In conclusion, the recently proposed nanoflake model provides a structural origin for the anomalous density–temperature relation of silica glass. The density anomaly of silica glass can be explained as a byproduct of the formation of a medium-range ordering structure in the glass transition and provides additional support for the new structural model.

## Data Availability

Data Availability Statements are available in section “MDPI Research Data Policies” at https://www.mdpi.com/ethics.
